# Recommendations for digital phenotyping using consumer grade wearable devices among people with severe mental illness: lessons from a systematic review of the literature

**DOI:** 10.1192/j.eurpsy.2025.521

**Published:** 2025-08-26

**Authors:** L. Hassan, C. Sawyer, A. Milton, J. Torous, A. J. Casson, A. Davies, B. Ruiz-Yu, J. Firth

**Affiliations:** 1 University of Manchester, Manchester, United Kingdom; 2 University of Sydney, Sydney, Australia; 3 Harvard Medical School, Boston, United States

## Abstract

**Introduction:**

Digital wearable devices, such as smartphones and smartwatches, have shown potential for passively monitoring mental and physical health in individuals with Severe Mental Illness (SMI), such as schizophrenia and bipolar disorder. While research-grade devices are well studied, consumer-grade wearables could offer a more accessible alternative, though their utility in this specific context remains underexplored.

**Objectives:**

We conducted a systematic review to assess the utility of data from consumer-grade wearables in tracking and predicting changes in mental and physical health among adults with SMI. We focused on passively collected physiological data, such as sleep patterns, physical activity, and heart rate. We sought to a) identify relationships between data streams and both mental and physical health outcomes and b) recommendations for future digital phenotyping research.

**Methods:**

A systematic review of multiple databases (Cochrane Central Register of Controlled Trials, APA PsycINFO, Embase, MEDLINE, and IEEE XPlore) was conducted in May 2024. Studies that collected passive physiological data for at least three days were included. Narrative methods were used to synthesise results across three key phenotypes: physical activity, sleep and circadian rhythms, and heart rate. Studies using invasive, or research-specific devices were excluded.

**Results:**

In total, 23 studies met the inclusion criteria, representing data from 12 distinct studies and more than 500 participants with SMI, mostly from high-income countries. The majority of studies used smartphones (N=15), with only eight utilizing smartwatches or other wrist-worn wearables. Eighteen studies focused on physical activity, 14 on sleep and/or circadian rhythms, and six on heart rate. We explore the findings of this study, focusing on practical recommendations for future research in the following areas: exploiting opportunities to promote physical health outcomes in SMI; greater standardization of reporting and methodologies; fine tuning longitudinal data collection and feature definition; and comparing alternative data analysis strategies.

**Conclusions:**

Consumer-grade wearables hold significant promise for the passive monitoring of both mental and physical health in individuals with SMI, though current research focuses largely on psychiatric relapse prevention. The findings of this systematic review provide insights into research gaps and future research directions, including tackling physical comorbidities in this population.

**Disclosure of Interest:**

L. Hassan: None Declared, C. Sawyer: None Declared, A. Milton: None Declared, J. Torous Grant / Research support from: Otsuka , Consultant of: Precision Mental Wellness, A. Casson: None Declared, A. Davies: None Declared, B. Ruiz-Yu: None Declared, J. Firth Consultant of: Atheneum, Informa, Bayer, HedoniaUSA, Strive Coaching, Angelini, ParachuteBH, and the Richmond Foundation.

